# Evaluation of Subclinical Hand Joint Synovitis by Ultrasonography in Patients with Psoriatic Arthritis and Cutaneous Psoriasis: A Case-Control Study

**DOI:** 10.31138/mjr.33.4.421

**Published:** 2022-12-31

**Authors:** Sumantro Mondal, Rudra Prosad Goswami, Debanjali Sinha, Geetabali Sircar, Arpita Hati, Debasish Lahiri, Parasar Ghosh, Alakendu Ghosh

**Affiliations:** 1 Department of Clinical Immunology and Rheumatology, Institute of Post Graduate Medical Education and Research, Kolkata, India,; 2 Department of Rheumatology, All India Institute of Medical Sciences, New Delhi, India,; 3 Department of Dermatology, Institute of Post Graduate Medical Education and Research, Kolkata, India

**Keywords:** psoriatic arthritis, cutaneous psoriasis, subclinical synovitis, ultrasonography

## Abstract

**Objectives:**

To find the frequency of subclinical hand joint synovitis (SS) in patients with psoriatic arthritis (PsA) and cutaneous psoriasis (PsC) compared to healthy controls (HC), and correlation of SS with disease activity.

**Methods:**

PsA patients (n= 52), without any past/current history of hand joint arthritis, PsC patients (n= 48), and 45 HC were recruited. Grey-scale and power Doppler Ultrasonography of bilateral hand joints were performed. The proportions of hand joints with SS were estimated in each group. A wrist-ray score was devised. Correlations were obtained between the number of joints with SS and disease activity parameters.

**Results:**

Higher proportion of PsA patients (55.8%) had SS than PsC (29.2%, p= 0.007), and HC (22.2%, p=0.001). Proportion of joints with SS was higher in PsA patients (5.38%) compared to PsC (2.92%, [p=0.0008]), and HC (1.11%, [p=0.0007]). Compared to HC, PsA patients had significantly higher bilateral ray 3 (p=0.002 and 0.01 for left and right ray 3, respectively), and right ray 4 involvement (p=0.037) and PsC patients had higher left ray 3 involvement (p=0.03). Wrist-ray score above 2.5 could distinguish patients of PsC with significant subclinical synovitis compared to controls (area under curve: 0.857, 95% confidence interval: 0.71–1.00). There was a significant correlation of SS with ESR in PsA group (p-value: 0.044), and with CRP in PsC group (p-value: 0.003), but not with other disease activity indices.

**Conclusion:**

SS was noted in approximately half of PsA and 1/3^rd^ of PsC patients. Both PsA and PsC patients had a significantly higher number of hand joint SS than HC. Ray pattern of hand joint SS could be present in both PsA and PsC.

## INTRODUCTION

Psoriatic arthritis (PsA) is an inflammatory arthritis, which is usually associated with cutaneous psoriatic lesions. In addition to the involvement of skin and joint, PsA patients also suffer from various extra-articular manifestations. In the majority of patients, cutaneous Psoriasis (PsC) appears before the joint involvement. PsA is associated with significant joint erosion and radiographic structural damage. Structural damage can be seen in nearly half of the PsA patients within two years of symptom onset.^1^ In one study, 58% of patients with PsA had radiologic damage at baseline which increased to 74% at 5 years follow-up.^2^ Due to the progressive radiological damage, PsA causes significant disability if not treated optimally at an earlier stage.

Patients with PsC are at increased risk for the development of PsA during their lifetime.^3^ The reported prevalence of the development of PsA among patients with PsC shows a wide variation ranging from 2–26% however, a higher prevalence rate of up to 48% have also been reported.^4,5^

Subclinical synovitis is a relatively newer concept in Rheumatology. It denotes the detection of joint inflammation by advanced imaging techniques in clinically normal joints in patients with inflammatory arthritis. Ultrasonography (USG) and Magnetic Resonance Imaging (MRI) are the most commonly used imaging modalities for the detection of subclinical synovitis. Mostly studied in patients with Rheumatoid arthritis (RA), it has been shown that subclinical synovitis can be present in a significant proportion of RA patients, even in the clinical remission state.^6^ It is worth mentioning that, the persistence of subclinical synovitis can be predictive of RA disease flare and radiographic progression.^7–9^ Similarly, patients with PsA may have subclinical synovitis, and in one study, 75% of early PsA patients had subclinical synovitis. Wrist, knee, and metacarpophalangeal joints were the most common sites for subclinical synovitis.^10^ Importantly, power Doppler (PD) USG detected synovitis may also predict short-term flare in the PsA patients who are in remission state.^11^ The presence of various subclinical articular and peri-articular inflammatory lesions has been shown among patients with PsC.^12–15^ Notably, the presence of subclinical synovitis or enthesitis in patients with PsC can predict the future development of PsA.^16,17^

It has been recently proposed that the transition of PsC to PsA occurs through clinically quiescent phases and more importantly, the possible beneficial role of Interleukin 17A inhibition in PsC patients with subclinical inflammatory changes have also been shown.^18,19^ Most of these studies compared either PsA or PsC patients with healthy controls. Comparative evaluation of subclinical synovitis among patients with PsA, PsC, and controls from the same population cluster has rarely been done previously.^20,21^ Indian data regarding this issue is also lacking.

With this background, this study was intended to evaluate the frequency and distribution of subclinical hand joint synovitis by USG in patients with PsC and early PsA in comparison to healthy controls. Any possible correlation between PsA and PsC disease activity measures and subclinical synovitis was also assessed.

## MATERIALS AND METHODS

This cross-sectional study was approved by the Institute of Post Graduate Medical Education and Research, Kolkata, India research oversight committee. Patients with PsA (≤ 2 years of disease duration), without any clinical evidence of hand joint involvement, were recruited from the outpatient clinic of the Department of Clinical Immunology and Rheumatology of our institution. The diagnosis of PsA was confirmed by a Rheumatologist using the CASPER classification criteria, 2006.^22^ PsC patients (diagnosed by a Dermatologist clinically or biopsy-proven Psoriasis) were recruited from the Department of Dermatology of the same institution. All the participants of this study had an age range between ≥ 18 to ≤ 45 years. Written informed consent was obtained from each participant. The following exclusion criteria were used during patient selection: 1. Pregnancy; 2. Obesity; 3. Diabetes mellitus or impaired glucose tolerance; 4. Treatment with systemic corticosteroids in the past four weeks; 5. Hypothyroidism; 6. Patients with other coexistent rheumatological diseases; 7. Current or past history of any biologic disease-modifying anti-rheumatic drug use; and 8. PsC patients with a current or past history of oral small molecule (OSM: Methotrexate, Sulfasalazine, Leflunomide, Cyclosporine, and Apremilast) intake. Age and sex-matched healthy volunteers were included for comparison. Baseline demographic and clinical data were collected. Disease Activity in Psoriatic Arthritis (DAPSA) Score and Psoriasis Area and Severity Index (PASI) score was calculated in patients with PsA, and the PASI score was calculated in patients with PsC. DAPSA score was calculated by a Rheumatologist and the PASI score by a Dermatologist. Blood samples were collected from the PsA and PsC patients. A complete hemogram including Erythrocyte sedimentation rate (ESR) was estimated. hs-C-reactive protein (hs-CRP) was measured by nephelometry.

### Detection of subclinical synovitis by Ultrasonography

**Figure 1.**
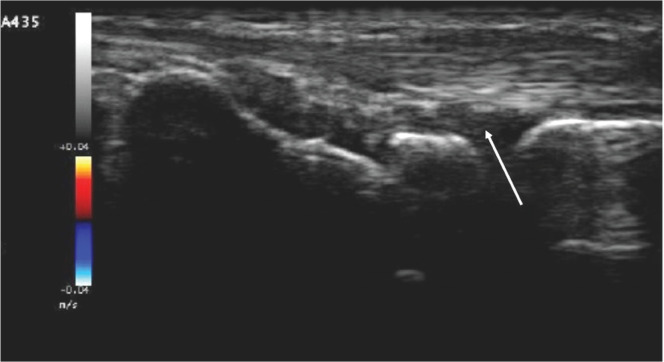
Representative image of grade 2 synovial proliferation of the wrist joint (white arrow).

The procedure was performed by a radiologist experienced in musculoskeletal (MSK) USG and was blinded to the clinical and laboratory details of the participants. My Lab 25 Gold USG platform was used for this purpose. MSK setting (wrist) was used for the assessment of hand joints. A high-frequency linear array transducer with a frequency range of 12–18 MHz was used. Synovial proliferation and vascularity were assessed in Greyscale (GS) and power Doppler (PD), respectively. A low pulse repetition frequency (0.5–0.7 KHz) was used for PD to optimize the detection of slow flow synovial vessels. Colour gain and filter were adjusted to exclude background noise from static structures and artifacts. In each patient, 15 joints of each hand (total 30 joints) were scanned on the same day of clinical assessment and blood sample collection. The following hand joints were assessed bilaterally; wrist, 1^st^ to 5^th^ metacarpophalangeal (MCP) joints, 1^st^ to 5^th^ proximal interphalangeal (PIP) joints, and 2^nd^ to 5^th^ distal interphalangeal (DIP) joints. Each joint was scanned from both the dorsal and palmar planes in the longitudinal and transverse axis. In each joint, greyscale (GS) synovial hypertrophy and PD vascularity were graded on a 0–3 scale as per recommendation.^23^ Sub-clinical synovitis was defined by the presence of a GS score ≥ 2 and/or a PD score ≥ 1 in any joint. A representative image of grade 2 synovial proliferation of the wrist joint is given in **Figure 1**.

### Statistical analysis

Linear data were expressed as mean ± standard deviation (SD) or median (interquartile range [IQR]), and categorical data as frequency (percentage). Deviation from normal distribution was tested with the Shapiro-Wilk test. Comparison of means was done with Mann Whitney U test and comparison of proportion with the Chi-Squared test or Fisher’s exact test as appropriate. We compared both the average number of positive joints per patient per group among the three groups and the total number of positive joints among all tested joints per group among the three groups. We also compared the USG positivity of the finger ray among all tested rays per group among the three groups. A ray was considered positive if any of the MCP, PIP, or DIP of a given ray had at least one positive joint. Finally, a linear scale was constructed combining all the GS and PD scores of the joints which afforded maximum discriminatory ability to distinguish PsA and PsC from the healthy adults. The cut-off of this score that best discriminates PsC from controls was determined using the Receiver operating characteristic curve. A p-value of ≤ 0.05 was considered statistically significant. All statistical calculation was done with SPSS ver. 21 (IBM Corp., USA).

## RESULTS

Overall, 145 subjects were studied: 52 patients with PsA [median age 33 years, (IQR: 27–38), 35 (67.3%) males, median duration of disease 14.5 months (IQR: 10–18)], 48 patients with PsC (median age 34.5 years, [IQR: 29–39], 32 [66.7%] males, median duration of disease 60 months [IQR: 48–84]) and 45 healthy controls (median age 31 years, [IQR: 25–35], 30 [66.7%] males). Among the patients with PsA, 55.8% (29/52) had subclinical synovitis which was significantly higher than the PsC patients (29.2% [14/48], p-value: 0.007), and significantly higher than the control subjects as well (22.2% [10/45], p-value: 0.001). No significant difference was observed between the PsC and the control group in this respect (p-value: 0.44). (Detailed description is given in **Table 1**).

**Table 1.** Clinical and USG parameters of the entire cohort.

**Parameters**	**Control n=45**	**PsC n=48**	**PsA n=52**	**p-value^a^**	**p-value^b^**	**p-value^c^**
Age in years	31 (25–35)	34.5 (29–39)	33 (27–38)	0.42	0.12	0.017
Male gender	30 (66.7)	32 (66.7)	35 (67.3)	0.95	0.95	1.00
Duration of disease in months	NA	48 (24–60)	14.5 (10–18)	<0.001	NA	NA
PASI	NA	9.4 (6.1–12.5)	5.1 (2.8–8.5)	<0.001	NA	NA
DAPSA	NA	NA	19.4 (11.5–23.6)	NA	NA	NA
Number of patients with any evidence of subclinical synovitis	10 (22.2)	14 (29.2)	29 (55.8)	0.007	0.001	0.44
Median number of positive joints per patient among patients with subclinical synovitis	1 (1–2)	3 (2–4)	2 (2–4)	0.84	0.009	0.016
Number of joints with subclinical synovitis	15/1350	42/1440	84/1560	0.0008	0.0007	0.0007

a p-value comparing PsA vs PsC,

b p-value comparing the groups PsA vs control.

c p-value comparing the groups PsC vs Control.

Cells designate either median (interquartile range) or number (%).

DAPSA: Disease Activity in PSoriatic Arthritis; NA: not applicable; PASI: Psoriasis Area Severity Index; PsA: Psoriatic arthritis; PsC: Cutaneous psoriasis; USG: Ultrasonography.

### Subclinical synovitis among the three groups

Overall, 4350 joints were scanned: 1560 among patients with PsA, 1440 among patients with PsC, and 1350 among controls. Frequency of positivity (GS≥2 and/or PD≥1) was highest among patients with PsA (84 [5.38%, 95% CI: 4.37–6.62]) followed by PsC (42 [2.92%, 95% CI: 2.17–3.92]) and controls (15 [1.11%, 95% CI: 0.67–1.83]). The number of joints with subclinical synovitis in the PsA group was significantly higher than the PsC group (p-value: 0.0008), and controls (p-value: 0.0007). PsC patients had a significantly higher number of joint involvement than controls (p–value: 0.0007).

In patients with PsA, only GS positivity was noted in 56 joints ([66.66%, 95% CI: 56.05 – 75.82, of the joints with subclinical synovitis] and [3.58%, 95% CI: 2.77–4.63, of the total joints, scanned]). PD positivity, with or without GS positivity was noted in 28 joints ([33.33%, 95% CI: 24.18 – 43.95, of joints with subclinical synovitis] and [1.79%, 95% CI: 1.24–2.58, of total joints, scanned]).

In PsC patients, only GS positivity noted in 29 joints ([69.04%, 95% CI: 53.97–80.93, of the joints with subclinical synovitis] and [2.01%, 95% CI: 1.40 – 2.87, of the total joints scanned]) and PD positivity, with or without GS positivity was noted in 12 joints ([28.57%, 95% CI: 17.17 – 43.57, of the joints with subclinical synovitis] and [0.83%, 95% CI: 0.47–1.45, of the total joints, scanned]). The median numbers of joints showing subclinical synovitis per patient were significantly higher in both PsA and PsO groups compared to controls (**Table 1**).

Patients with PsA had more affliction of the bilateral wrist and DIP3 joints compared to controls. Among the total 104 wrist joints scanned in PsA patients 32 joints (30.76%, 95% CI: 22.72–40.19) were positive for subclinical synovitis followed by the DIP3 (13/416 (3.12%, 95% CI: 1.83–5.22]), DIP2 (6/416 [1.44%, 95% CI: 0.66–3.11], and MCP3 (6/520 [1.15%, 955 CI: 0.52–2.49]) joints. Among the total 416 DIP joints scanned 24 joints (5.76%, 95% CI: 3.90–8.44) had evidence of subclinical synovitis. Patients with PsC had a higher frequency of left wrist joint involvement compared to controls. In this group, subclinical synovitis was most frequently noted in wrist joints (20/96 [20.83%, 95% CI: 13.91–30.0]) followed by DIP3 (5/384 [1.30%, 95% CI: 0.55–3.01]) and DIP2 (4/384 [1.04%, 95% CI: 0.40–2.64]) joints. In the PsC patients, 12 DIP joints in total (12/384 [3.12%, 95% CI: 1.79–5.38]) showed evidence of subclinical synovitis.

The joint-specific distribution of subclinical synovitis among three study groups is given in **Table 2**.

**Table 2.** Joint level distribution of subclinical synovitis among the three groups.

**Left**				**Right**							
**Joints**	**PsA n=780**	**PsC n=720**	**Controls n=675**	**Joints**	**PsA n=780**	**PsC n=720**	**Controls n=675**
MCP1	2	0	1	MCP1	2	1	0
MCP2	2	0	1	MCP2	1	0	0
MCP3	3	1	0	MCP3	3	0	0
MCP4	1	1	0	MCP4	0	1	0
MCP5	1	1	0	MCP5	0	0	0
PIP1	1	1	0	PIP1	0	0	1
PIP2	1	0	0	PIP2	4	0	0
PIP3	1	1	0	PIP3	1	1	0
PIP4	1	1	0	PIP4	3	0	0
PIP5	1	0	0	PIP5	0	1	1
DIP2	3	3	0	DIP2	3	1	1
DIP3	7^a^	3	0	DIP3	6	2	1
DIP4	2	2	0	DIP4	2	0	0
DIP5	1	0	0	DIP5	0	1	0
WJ	15^a^	11^b^	3	WJ	17^c^	9	6
Ray 1	3	1	1	Ray 1	2	1	1
Ray 2	6	3	1	Ray 2	8	1	1
Ray 3	11^d^	5^e^	0	Ray 3	10^f^	3	1
Ray 4	4	4	0	Ray 4	5^g^	1	0
Ray 5	3	1	0	Ray 5	0	2	1

a p-value between PsA vs control: 0.01,

b p-value between PsC vs control: 0.04,

c p-value between PsA vs control: 0.047,

d p-value between PsA vs control: 0.002,

e p-value between PsC vs control: 0.03,

f p-value between PsA vs control: 0.01,

g p-value between PsA vs control: 0.037

DIP: distal inter-phalangeal joint; MCP: metacarpophalangeal joint; PIP: proximal interphalangeal joint; PsA: Psoriatic arthritis; PsC: Cutaneous psoriasis; WJ: wrist joint.

**Figure 2.**
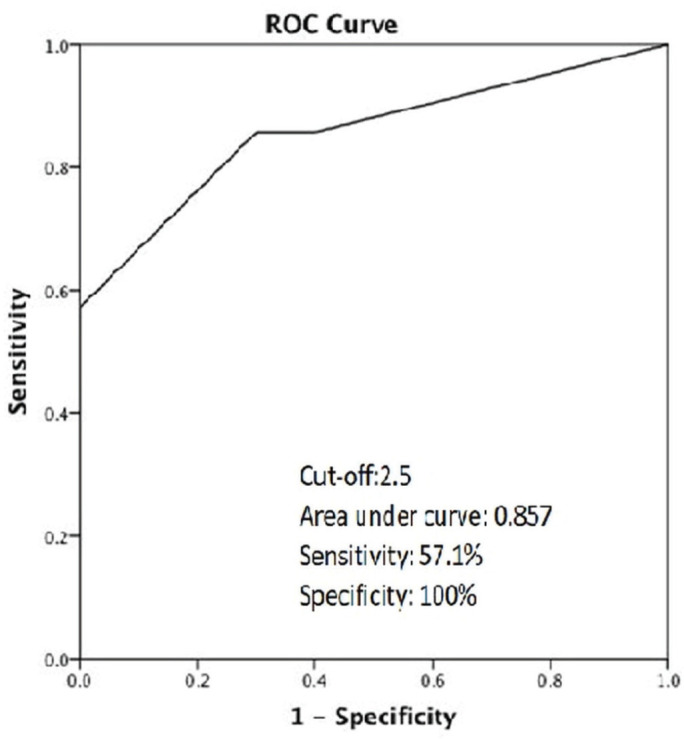
Receiver operating characteristic curve showing the cut-off value of Ray-Wrist score between patients of PsC with any subclinical synovitis and controls.

In terms of ray involvement, compared to the control population, patients with PsA had significantly higher bilateral ray 3 (p-value: 0.002 and 0.01 for left and right ray 3, respectively), and right ray 4 involvement (p-value: 0.037), and patients with PsC had higher left ray 3 involvement compared to controls (p-value: 0.03). The sum of left and right ray 4 involvement among patients with PsC was higher compared to controls (5/1440 vs 0/1350, p=0.03). We devised a linear scale combining all GS and PD scores of MCP (3, 4), PIP (3, 4), DIP (3, 4), and left wrist joint (Ray-Wrist score). The distribution of the Ray-Wrist score is given in **Table 3**. ROC curve was drawn to determine the cut-off value of the Ray-Wrist score between patients of PsC with any subclinical synovitis and controls (**Figure 2**). A score above 2.5 could distinguish patients of PsC with significant subclinical synovitis compared to controls (Area under curve 0.857, 95% CI: 0.71–1.00, sensitivity: 57.1%, specificity: 100%).

**Table 3.** Distribution of the Ray-Wrist score among the three groups of subjects.

		**Control n=45**	**PsC n=48**	**PsA n=52**	**p-value^a^**	**p-value^b^**	**p-value^c^**
Ray-Wrist Score	Mean ± SD	0.16 ± 0.52	1.1 ± 2.23	1.87 ± 2.37	0.023	<0.001	0.025
	95% CI	0–0.31	0.44–1.76	1.2–2.52			

a P-value comparing PsA vs PsC

b P-value comparing the groups PsA vs control

c P-value comparing the groups PsC vs Control

CI: confidence interval; PsA: Psoriatic arthritis; PsC: Cutaneous psoriasis; SD: standard deviation.

### Association of subclinical synovitis with disease activity measures

A comparison of the presence of subclinical synovitis and disease activity measures is given in **Table 4**. PsC patients with subclinical synovitis had significantly higher CRP than patients without (p-value: 0.003). In the PsA group, patients with subclinical synovitis had significantly higher ESR levels compared to those without (p-value: 0.044). Positive CRP (>3mg/dL) was numerically frequent among patients with PsC with subclinical synovitis (respectively, 5/14 [36%] vs 5/34 [15%], p=0.13) but was equally common among patients with PsA with or without subclinical synovitis (respectively, 6/29 [21%], vs 9/23 [39%], p=0.15). PsA patients with subclinical synovitis and high CRP level had a higher number of joints showing subclinical synovitis. PsA patients with or without subclinical synovitis did not show any significant difference in DAPSA and PASI score, and the PASI score was not significantly different between PsC patients with or without subclinical synovitis (**Table 4**).

**Table 4.** Association of subclinical synovitis with disease activity parameters among patients with PsC and PsA.

**Parameters**	**PsA**			**PsC**		
	**Subclinical synovitis**		**p-value**	**Subclinical synovitis**		**p-value**
	Present n=29	Absent n=23		Present n=14	Absent n=34	
ESR in mm/hr	33.5 (21–45.5)	23 (13–33)	0.044	30 (22.5–36.5)	25.5 (17.8–34)	0.38
CRP in mg/dL	1.3 (0–2.7)	1.8 (0–3.1)	0.58	2.5 (1.6–3.2)	0.45 (0–1.94)	0.003
DAPSA	19.8 (10.8–24)	19.6 (13–23.7)	0.692	NA	NA	
PASI	5.3 (2.3–9.9)	5.1 (3.4–6.7)	0.71	10.2 (6.4–13.6)	9.2 (5.6–11.9)	0.49
CRP<3mg/dL	2 (1–4)*	NA	0.045	2 (2–5)*	NA	0.68
CRP≥3mg/dL	4 (3–5)*	NA		3 (2–6)*	NA	

* Number of joints with subclinical synovitis

CRP: C-reactive protein; DAPSA: Disease Activity in PSoriatic Arthritis; ESR: erythrocyte sedimentation rate; NA: not applicable; PASI: Psoriasis Area Severity Index; PsA: Psoriatic arthritis; PsC: Cutaneous psoriasis.

## DISCUSSION

PsA patients with a disease duration of ≤ 24 months were recruited for this study whose hand joints were clinically normal. Only hand joints were evaluated in our study as these joints are commonly affected in the PsA disease course.^24^ Furthermore, hand joint involvement may be associated with a higher functional impairment in PsA.^25^ In our study, PsC patients did not have any clinical evidence of synovitis in any joints. Approximately, 56% of the PsA patients in our study showed evidence of subclinical synovitis in hand joint/s scanned by USG, which is significantly more compared to the PsC and control group. One previous study reported a higher proportion of PsA patients with subclinical synovitis.^10^ In a study by Freeston JE et al., 96% of the DMARD-naïve, recent-onset PsA patients had evidence of subclinical synovitis, which was reduced to 75% if MTP and ankle joints were excluded. They also reported a higher proportion of joints (16.6%) showing subclinical synovitis overall, and in 13.5% of joints excluding MTP and ankle joints, which is more than two-fold compared to our finding (5.38%). It is noteworthy that, only DMARD-naïve PsA patients were included by Freestone JE et al., whereas ongoing OSM treatment in PsA patients was not an exclusion criterion in our study. They also scanned more joints (40 joints/patient) including shoulder, elbow, knee, tibiotalar, and MTP joints. The inclusion of additional joints could be an explanation for the detection of a higher proportion of subclinical synovitis in their study. Regarding the proportion of joints with PD positivity, our data (1.8%) did not show much difference from them (2.5%). The wrist joint was the most common to show subclinical synovitis in both the studies in an almost similar frequency (approximately 30%). Following the wrist joint, DIP joints were the 2^nd^ most common to show subclinical synovitis (5.77%) in our study. DIP joint involvement is one of the characteristic features of PsA however, it was not assessed by Freestone JE et al.^10^

The proportion of PsC patients with subclinical synovitis in our study was not significantly different from the control population. The proportion of joints with subclinical synovitis in the PsC group was however significantly more than the healthy controls. The frequency of joint involvement in PsC followed a similar trend to the patients with PsA (wrist>DIP3>DIP2). Some of the previous studies documented subclinical synovitis among patients with PsC without any musculoskeletal manifestations.^12,16,17^ In a recent study by Zuliani F et al. subclinical synovitis was noted in 27.5% of the Psoriatic patients, which is almost equal to our finding (29.2%). However, when they excluded MTP joints from their analysis the proportion was further curtailed to 20%.^26^ In this regard, our data is also corroborative with one Saudi Arabian study which reported subclinical synovitis in 31% of the patients with psoriasis.^17^ One study however documented a higher prevalence of subclinical synovitis in patients with PsC. In this study with 136 patients with psoriasis, Naredo E et al. showed subclinical synovitis in almost 50% of the participants.^12^ In all these studies, USG was used to detect subclinical synovitis. Faustini F et al. reported subclinical synovitis in 38% of the Psoriasis patients using MRI of the hand as the imaging modality.^16^ The proportion of joint with evidence of subclinical synovitis in our study (2.9%,) is slightly higher than that reported by Elnady B et al. (1.4%), and Zuliani F et al. (1.3%), albeit lower than that reported by Naredo E et, al. (3.2%).

A ray pattern of joint involvement has been described in PsA in which classically all three joints of a hand digit show evidence of inflammation.^27,28^ A less stringent definition of ray involvement was, however, adopted in our study, and subclinical synovitis in any joint of a particular digit was considered as ray involvement. In our study, bilateral ray 3 and right ray 4 involvement was significantly more common in PsA patients, than in the control subjects. PsC patients also showed a higher frequency of left ray 3 and summed bilateral ray 4 involvement than control. This finding was not addressed in previous studies. One recent study documented that a ray pattern may be more common in PsA joint activity than damage.^29^ In concordance with this finding, subclinical joint activity in PsA may also follow a ray pattern. Importantly, we devised a linear Ray-Wrist score and derived a cut-off value to differentiate PsC patients with subclinical synovitis from the healthy population. It will be too premature to come to a definite conclusion from these findings in a small number of patients and it requires further evaluations.

A cross-sectional study design is one of the limitations of our study. A prospective study with long-term follow-up would be more effective, as it could provide information about the development of clinically evident synovitis in the affected joints of PsA patients if any. Simultaneously, in PsC patients, the significance of subclinical synovitis could be assessed. The small sample size is another limitation of our study. Most of the PsA patients in our study were taking OSM with or without concomitant NSAIDs. These medications might have some impact on the USG findings in the PsA group.

Despite these limitations, our study showed the burden of subclinical synovitis among a sub-group of Indian PsA and PsC patients. To the best of our knowledge, this is the first Indian study regarding this issue and among the few global studies, which evaluated subclinical synovitis in PsA and PsC simultaneously. Our study also showed the ray pattern of involvement in digits which was not addressed before.
